# Vascular Co-Option and Other Alternative Modalities of Growth of Tumor Vasculature in Glioblastoma

**DOI:** 10.3389/fonc.2022.874554

**Published:** 2022-03-30

**Authors:** Domenico Ribatti, Francesco Pezzella

**Affiliations:** ^1^ Department of Basic Medical Sciences, Neurosciences and Sensory Organs, University of Bari Medical School, Bari, Italy; ^2^ Nuffield Division of Laboratory Science, Radcliffe Department of Medicine, University of Oxford, John Radcliffe Hospital, Oxford, United Kingdom

**Keywords:** angiotropism, glioblastoma, glioblastoma stem cells, vascular co-option, vasculogenic mimicry

## Abstract

Non-angiogenic tumors grow in the absence of angiogenesis by two main mechanisms: cancer cells infiltrating and occupying the normal tissues to exploit pre-existing vessels (vascular co-option); the cancer cells themselves forms channels able to provide blood flow (the so called vasculogenic mimicry). In the original work on vascular co-option initiated by Francesco Pezzella, the non-angiogenic cancer cells were described as “exploiting” pre-existing vessels. Vascular co-option has been described in primary and secondary (metastatic) sites. Vascular co-option is defined as a process in which tumor cells interact with and exploit the pre-existing vasculature of the normal tissue in which they grow. As part of this process, cancer cells first migrate toward vessels of the primary tumor, or extravasate at a metastatic site and rest along the ab-luminal vascular surface. The second hallmark of vascular co-option is the interaction of cancer cells with the ab-luminal vascular surface. The first evidence for this was provided in a rat C6 glioblastoma model, showing that the initial tumor growth phase was not always avascular as these initial tumors can be vascularized by pre-existing vessels. The aim of this review article is to analyze together with vascular co-option, other alternative mode of vascularization occurring in glioblastoma multiforme (GBM), including vasculogenic mimicry, angiotropism and trans-differentiation of glioblastoma stem cells.

## Canonical and Alternative Mode of Growth of Tumor Vasculature

**Figure 1:**
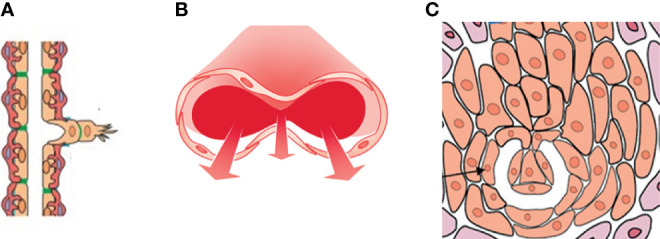
A drawing showing the three types of angiogenesis have been described in tumor growth: **(A)** sprouting angiogenesis, **(B)** intussusceptive microvascular growth (IMG), and **(C)** glomeruloid vascular proliferation. Sprouting angiogenesis involves formation and outgrowth of sprouts; IMG involves the formation of new vasculature where a pre-existing vessel splits in two; in glomeruloid vascular proliferation small glomeruloid bodies, so-called for their morphological resemblance with the renal glomeruli, are recognizable.

Three types of angiogenesis have been described in tumor growth: sprouting angiogenesis (1), intussusceptive microvascular growth (IMG) (2), and glomeruloid vascular proliferation (3) (**Figure 1**). Sprouting angiogenesis in tumor growth include the following stages: The basement membrane is locally degraded on the side of the dilated peritumoral postcapillary venule situated closed to the angiogenic stimulus; Interendothelial contacts are weakened and endothelial cells migrate into the connective tissue; A solid cord of endothelial cells form; Lumen formation occurs proximal to the migrating front, contiguous tubular sprouts anastomose to form functionally capillary loops, parallel with the synthesis of the new basement membrane and the recruitment of pericytes (1).

In IMG, the vascular network expands by insertion of newly formed columns of interstitial tissue structures (tissue pillars) into the vascular lumen. IMG proceeds through these steps: Protrusion of opposing capillary walls into the lumen and the creation of a contact zone between facing endothelial cells; Reorganization of their intercellular junctions and central perforation of the endothelial bilayer; Formation of an interstitial pillar core by invading supporting cells (myofibroblasts, pericytes) and deposition of matrix, such pillars ranging in diameter from 1 to 2.5 μm; Enlargement in thickness of the pillars without additional qualitative alteration (2). IMG occurs in different tumors, including colon and mammary carcinomas, melanoma, B-cell non-Hodgkin’s lymphoma and glioma (4).

A switch from sprouting to IMG might represent an adaptive response to treatment with various antitumor and anti-angiogenic compounds to restore the hemodynamic and structural properties of the vasculature enhancing tumor drug delivery and sensitivity to treatments (5).

**Figure 2:**
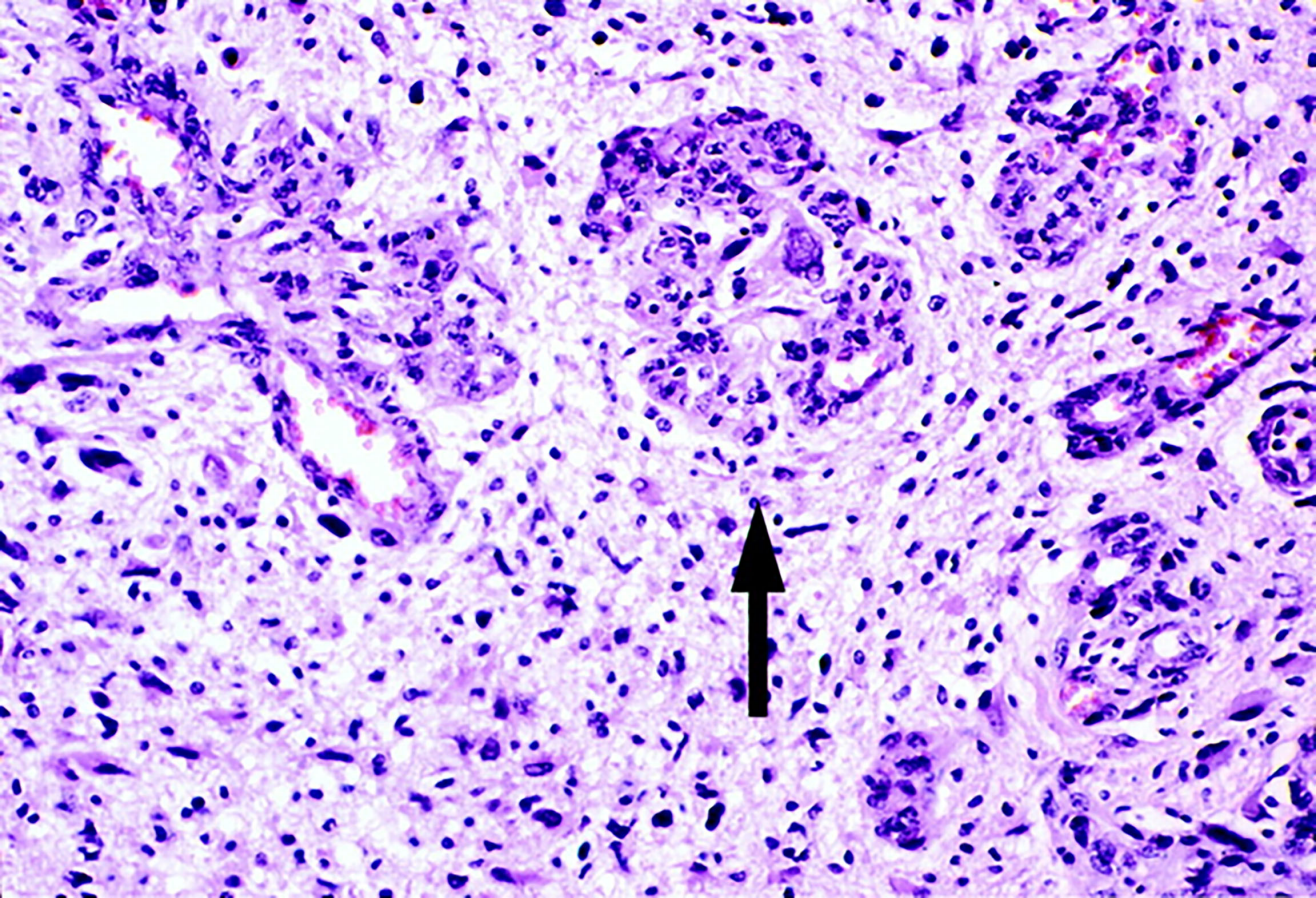
Glomeruloid vascular proliferation in a human glioblastoma multiforme bioptic specimen (arrow). Newly sprouted vessels arranged in tufted aggregates resemble renal glomeruli. Adjacent vessels demonstrate other morphological forms of microvascular hyperplasia in glioblastoma. Blue toluidine staining. Original magnification: x 25 (Reproduced from 7).

In glomeruloid vascular described in glioblastoma (6), small glomeruloid bodies, so-called for their morphological resemblance with the renal glomeruli, are recognizable (**Figure 2**). Glomeruloid bodies are made up by small vessels lined by hyperplastic endothelial cells surrounded by a discontinuous layer of pericytes. Two types of glomeruloid bodies might exist (6). The first, formed by an “active” mechanism would be the one in which angiogenesis occurs and the glomeruloid vessels are newly formed, possibly because of the action of vascular endothelial growth factor (VEGF) (3). The second type or “passive” is one in which no new vessels are formed but pre-existing capillaries are coiled and folded by metastatic cells which extravasate and then adhere to the abluminal surface of the capillaries and pulling them into a glomeruloid shape (6).

Tumors can also grow without inducing angiogenesis, as occurs in vessel co-option or vascular co-option (8), vasculogenic mimicry and angiotropism (9). In the original work on vascular co-option initiated by Francesco Pezzella, the non-angiogenic cancer cells were described as “exploiting” pre-existing vessels (10). Vascular co-option, described in primary and secondary (metastatic) sites, is defined as a process in which tumor cells interact with and exploit the pre-existing vasculature of the normal tissue in which they grow. In vessel co-option, tumors utilize alternative mechanisms besides angiogenesis to obtain nutrients for growth through local tumor invasion and proliferation along co-opted vessels. Cancer cells migrate along the pre-existing vessels and infiltrate tissues between co-opted vessels (8).

Vessel co-option was initially described in gliomas and lung metastasis (11–13). The first event observed following co-option was an increase in the levels of angiopoietin-2 (Ang-2) in the pre-existing vessels surrounded by tumor cells (11), without increase of VEGF expression, leading to vascular regression by detachment of the endothelium from the basement membrane. Ang-2 binds to its receptor Tie-2 inducing dissociation of the mural cells from endothelial cells (11). Moreover, Angiopoietin-2 (Ang-2) increases the secretion of matrix metalloproteinase-2 (MMP-2) favoring human glioma cells invasive capacity (14).

In vasculogenic mimicry, first described in uveal melanoma (15), tumor cells form vessel-like networks. In this condition, tumor cells reverse to an embryonic-like phenotype and mimic endothelial cells. Vasculogenic mimicry can serve as a marker for tumor metastasis, a poor prognosis, worse survival, and the highest risk of cancer recurrence.

Angiotropism (the pericytic-like location of tumor cells) is a microscopic marker of migration of tumor cells along the abluminal vascular surface (9). Glioma cells follow ab-luminal surface of blood vessels (16) and migrate considerable distances without employing intravascular dissemination (17).

## Vascularization of Glioblastoma Multiforme

Glioblastoma multiforme (glioblastoma IDH-wild type) is the most aggressive brain tumor with high recurrence and mortality rate. To further limit the molecular heterogeneity of tumors subsumed as ‘glioblastoma’, the upcoming 2021 World Health Organization (WHO) classification of primary brain tumors will introduce a definition of glioblastoma based on typical histological features and the absence of IDH mutations (18). IDH mutations characterize a subpopulation of glioblastomas and indicate a better prognosis (18). The vasculature of IDH mutated glioblastomas differs from that of IDH wild-type GBM, including a lower frequency of vascular abnormalities in IDH mutated glioblastomas (19).

With a median survival of 14-18 months and 5-year survival rates of less than 5%, the prognosis of GBM patients is very poor (20). The standard treatment for GBM patients is maximal tumor resection followed by adjuvant radiotherapy and adjuvant chemotherapy using alkylating agent temozolomide (the “stupp protocol”, 21).

One of the most significant features of GBM is the hypervascularity and there is a significant correlation between the degree of angiogenesis and prognosis (22). VEGF is highly expressed in GBM and is correlated with the grade of malignancy and prognosis (23, 24). Other angiogenic cytokines, including hepatocyte growth factor (HGF), fibroblast growth factor-2 (FGF-2), platelet derived growth factor (PDGF), Angs, and interleukin-8 (IL-8) are also up-regulated in GBM (24–27). In GBM, tumor-associated macrophages (TAMs) crosstalk with Treg cells to release pro-angiogenic and immune-suppressive VEGF (28).

GBM vessels are characterized by structural and functional abnormalities, including altered association between endothelial cells and pericytes, leading to chronic hyperpermeability, vessel leakage, poor vessel perfusion and delivery of nutrients (29). All these morphological characteristics contribute to hypoxia, interstitial fluid pressure and enhanced susceptibility to metastatic invasion (30). Furthermore, hypoxia-mediated up-regulation of pro-angiogenic factors secretion by inflammatory and tumor cells, enhance vascular abnormalities.

**Figure 3:**
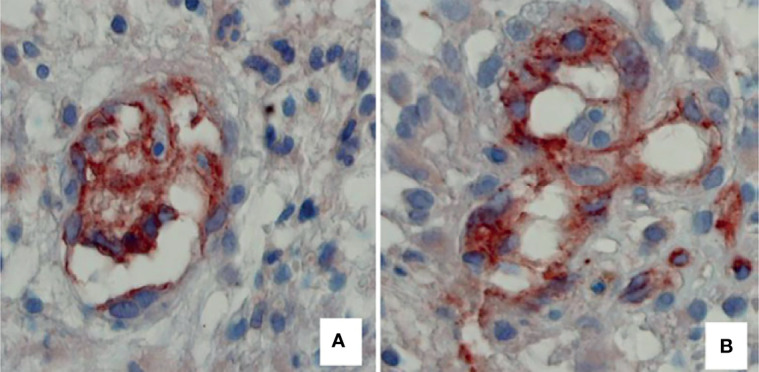
Two examples of tumor vessels, respectively, with a low and high number of connections of intraluminal tissue folds with the opposite vascular wall, expression of intussusceptive microvascular growth in II malignancy grade tumor specimen **(A)**, compared with IV malignancy grade **(B)**. Blood vessels have been identified by immunohistochemical reaction with an anti-CD31 antibody. Original magnification: x 60 (Reproduced from 4).

Different types of neovascularization occur in GBM, including vasculogenesis, angiogenesis, IMG (**Figure 3**), vascular co-option, vasculogenic mimicry, and trans-differentiation of glioblastoma stem-like cells (GSCs) in endothelial cell-like cells (31, 32). When GSCs were cultured *ex vivo* under endothelial favorable conditions, they expressed typical endothelial markers, such as CD31, von Willebrand factor (vWF), and Tie-2 (32, 33). Endothelial cells promote the GSC phenotype in the perivascular niche through direct cell–cell interactions by activating the Notch pathway in GSCs through the expression of Notch ligands and release of nitric oxide (34–37). Moreover, GSCs can secrete diffusible factors such as VEGF, which recruit tumor blood vessels to the niche (38, 39). Other modalities of interactions between tumor cells and endothelial cells in GMB include microRNA-containing extracellular vesicles, gap junctions and non-coding RNAs (40–43).

## Vascular Co-Option in Glioblastoma

C6 rat glioma cells co-opted brain vessels at early stages soon after their orthotopic injection (11). After serial transplantation of human derived GBM cells, early passaged tumor cells co-opted the brain vasculature, while at later passaged, angiogenesis occurs. Spheroids from human glioma patient tumors co-opt the host vasculature, showing an aggressive infiltrative growth pattern (44).

In GBM, tumor cells displace astrocytic endfeet from endothelial cells, leading to abnormal blood-brain barrier (BBB) permeability and loss of astrocytic-mediated glio-vascular coupling (17, 45–47). Caspani et al. (46) studied interactions occurring between GBM cells and pericytes associated with brain blood vessels and demonstrated that GBM cells produced cytoplasmic expansions denominated flectopodia which adhere to pericytes, forming hybrid cells.

## Other Alternative Mechanisms of Vascular Growth in Glioblastoma

Orthotopic injection of GSCs in immunocompromised mice generated large anaplastic tumor xenografts, showing a vessel wall formed by endothelial cells derived from GSCs (33). GSCs support vascular function by generating pericytes in a process enhanced by hypoxia (48). Endothelial cells induce GSCs features in differentiated GBM cells through FGF-2 (49), and tumor-derived endothelial cells share the same somatic mutations as GBM cells, suggesting that tumor endothelial cells derive from GMB cells (31).

In GBM, vasculogenic mimicry is characterized by the activation of epithelial-mesenchymal transition (EMT)-related proteins, such as Twist1 (50), up-regulation of IL-6 expression in glioma cells (51), and trans-differentiation of GSCs into mural cells (52).

## Resistance to Anti-Angiogenic Therapy in GBM

Resistance to anti-angiogenic treatment can be intrinsic, when it is observed at the beginning of the treatment, or acquired, i.e., that it affects the relapsing disease after an initial response to therapy (53).

Resistance to VEGF pathway inhibitors involves different mechanisms, including normalization of tumor blood vessels, alternative mechanisms of vessel formation, hypoxia, recruitment of inflammatory cells and immature myeloid cells (53). The most accepted hypothesis for acquired resistance to anti-angiogenic therapies is based on the induction or up-regulation of other pro-angiogenic factor pathways, including IL-8, FGF-2, PDGF and Angs (53). PDGF-BB can induce GBM formation when overexpressed with the RCAS system (54).

Non-angiogenic growth is an important mechanism of acquired resistance to anti-angiogenic therapy. Tumor cells might evade anti-VEGF therapies using existing vasculature and increasing the fraction of co-opted vessels (55). Vascular co-option has been proposed to be a mechanism of resistance to anti-VEGF therapies (56–58). In GBM, the aberrant vasculature favor increasing resistance and limitations to the efficacy of conventional therapies.

Anti-VEGF antibody treatment increased the fraction of co-opted vasculature in human glioblastoma cells injected into nude rat striatum (59). Treatment of GBM with a monoclonal antibody against VEGF receptor-2 (VEGFR-2) induces co-option of quiescent cerebral vessels (60). Modified GBM-resident endothelial cells express lower levels of VEGFR and this might ultimately dampen the efficacy of anti-VEGF therapies (61). Vascular co-option has been observed in GBM after anti-angiogenic therapy with cediranib (62).

Intravital imaging identified ephrin-B2 on endothelial cells and GSCs as an important regulator of vessel co-option and B11, a single-chain variable fragment directed against ephrin-B2 efficiently blocked cooption and tumor growth (13, 63). Chemotherapy and/or radiation therapeutic might increase GSC subpopulation and emerging tumor-derived endothelial cells. For instance, irradiated GSCs express Tie2, migrate towards VEGF, and form tubes on Matrigel *in vitro* (64). Moreover, temozolomide combined or not with bevacizumab, potentiates tumor-derived endothelial cell incorporation in vessels from xenograft models (65). In this context, GSC trans differentiation contributes to both resistance to anti-angiogenic therapies and re-vascularization following chemotherapy and/or radiation.

Bevacizumab obtained clinical approval by the US Food and Drug Administration for the treatment of GBM at progression after standard chemoradiotherapy. Bevacizumab inhibits angiogenesis and tumor growth in pre-clinical models of GBM (59, 66–68), and in combination with radiotherapy and chemotherapy with temozolomide was associated with a significant improvement of progression free survival (PFS), but only a modest improvement of overall survival (OS) (69–71). However, bevacizumab in combination with temozolomide or lomustine, respectively, did not prolong OS in patients with newly diagnosed or recurrent GBM in phase III clinical trials (71–73).

Several tyrosine kinase inhibitors, which inhibit PDGF receptor (PDGFR) and transforming growth factor beta (TGFβ), were ineffective in clinical trials (74–76). Chemotherapeutic stress after temozolomide treatment increase HIF response in recurrent GBM, leading to trans-differentiation of GSCs to endothelial cells, promoting vasculogenic mimicry (77).

Immune check-points inhibitors might induce an improved immune response against the co-opting cancer cells and might synergize with anti-angiogenic therapies (78). Immune check-points inhibitors have been successfully used in GBM mouse models (79–83), while immunotherapy is not working in human glioblastomas (84)”.

Blockade of VEGF, Ang-2, and PD-1 increased the survival of GBM-bearing mice in comparison to anti-VEGF and anti-Ang-2 alone (85). Targeting endothelial PAK4 promoted GBM vessel normalization, which in turn improved engineered chimeric antigen receptor T cells (CAR-T) infiltration and extended mouse survival (86).

## Author Contributions

DR conceived and wrote the manuscript; FP revised the manuscript. All authors contributed to the article and approved the submitted version.

## Funding

This work was supported in part by HORIZON EUROPE, GRANT CODE S08 (INTERGLIO) funded by the University of Bari Aldo Moro, Bari, Italy.

## Conflict of Interest

The authors declare that the research was conducted in the absence of any commercial or financial relationships that could be construed as a potential conflict of interest.

## Publisher’s Note

All claims expressed in this article are solely those of the authors and do not necessarily represent those of their affiliated organizations, or those of the publisher, the editors and the reviewers. Any product that may be evaluated in this article, or claim that may be made by its manufacturer, is not guaranteed or endorsed by the publisher.
